# Human Rights and Mental Health of Older Women

**DOI:** 10.1192/j.eurpsy.2022.125

**Published:** 2022-09-01

**Authors:** G. Stoppe

**Affiliations:** University of Basel, Mentage, Basel, Switzerland

**Keywords:** societal contribution, poverty, older women, ageism

## Abstract

Older persons represent a large, and the fastest growing, segment of the global population. Women form the majority of older persons with global demographic data consistently showing that women tend to live longer than men, especially at advanced ages. Older women also make substantial contributions to our societies and economies, including as informal caregivers, volunteers and community leaders. Ageing, however, is not gender-neutral and inequality and discrimination experienced by women during their lifespan is often exacerbated in older age. For example, older women are at a higher risk of living in poverty and of facing barriers in accessing basic rights such as health, adequate housing, and protection from violence, abuse and neglect. Yet, the specific challenges created by the intersection between age and gender often remain invisible and understudied. This also applies to people living in Europe. And elderly women with mental disorders face a triple stigma: suffering from mental disorders, being a woman, being old. The presentation emphasises the UN-decade of healthy ageing with fight against ageism, and a paper of the WHO and IPA on the topic of this presentation.

**Disclosure:**

No significant relationships.

